# O04 Orbital lymphoma in a 72-year-old lady with rheumatoid arthritis: an argument for rituximab

**DOI:** 10.1093/rap/rkab067.003

**Published:** 2021-10-19

**Authors:** Kieran Sandhu, Shabnam Shabbir, Alan Steuer

**Affiliations:** Rheumatology Department, Wexham Park Hospital, Frimley Health NHS Foundation Trust, Slough, United Kingdom

## Abstract

**Case report - Introduction:**

Rheumatoid Arthritis is associated with an increased risk of lymphoma.  The relative contribution of cumulative disease activity versus immunosuppressive treatments, in particular anti-TNF, to the overall risk remains controversial.  Awareness of this complication is important as most such lymphomas are highly treatment-sensitive when diagnosed early.

We report a case of orbital mucosa-associated lymphoid tissue (MALT) lymphoma in a patient with established rheumatoid arthritis with secondary Sjogren’s syndrome with quiescent disease treated with methotrexate and etanercept for many years. We discuss the risk factors, clinical signs and rationale for subsequent treatment decisions, including consideration of rituximab to treat both conditions.

**Case report - Case description:**

A 72-year-old lady with longstanding rheumatoid arthritis and secondary Sjogren’s reported a 1-year history of unilateral painless left watering eye and lid swelling. Vision was unaffected. There was no systemic upset or B-symptoms.  Her rheumatoid arthritis was quiescent on methotrexate and etanercept. Past medical history consisted of asthma, hiatus hernia, subtalar arthrodesis and granuloma annulare. Other medications included folate, naproxen, lansoprazole, rosuvastatin and salbutamol and beclomethasone inhalers. She was a current smoker with a 40 pack-year history. On examination, there was a palpable mass on the nasal aspect of her left upper eyelid.  Ocular examination was otherwise unremarkable and she had no lymphadenopathy.

Inflammatory markers were normal: CRP 2 mg/L and ESR 28 mm/hr with unremarkable full blood count, urea and electrolytes and liver function tests. LDH of 252 IU/L was at the upper limit of normal. Gadolinium-enhanced MRI revealed a 38mm x 20mm x 8mm soft tissue mass within the extraconal compartment of the left orbit, raising the possibility of lymphoma. CT chest, abdomen and pelvis showed no distant metastases.  Orbital biopsy revealed evidence of a kappa-restricted low-grade B-cell lymphoma with morphology and immunophenotype in keeping with MALT lymphoma.

The MDT decision was to actively monitor with interval re-imaging. Etanercept and low-dose methotrexate were continued. Follow-up MRI at 4 months revealed near complete resolution of the left orbital mass.  Her ocular symptoms had also spontaneously resolved. Etanercept was stopped at this time due to concerns around recent malignancy, with a plan to switch to rituximab. Her joints flared after stopping etanercept, but the first cycle of rituximab was very effective in controlling her arthritis. The patient is now awaiting a second cycle after a flare 9 months later.  She has had no recurrence of ocular symptoms and remains under close and active surveillance for lymphoma.

**Case report - Discussion:**

This case illustrates the increased risk of non-Hodgkin’s lymphoma in rheumatic disease, which may relate to impaired T-cell function in chronically active disease. MALT lymphomas account for around 8% of these; they originate from B cells and can affect nearly any mucosal site. Both chronic inflammation and immunosuppressive treatment contributed to increased risk here. Tumours are usually low-grade with onset in the 60s and 70s, with localised symptoms but minimal constitutional upset. Orbital MALT lymphomas present with a range of ocular symptoms; however, pain is rare and vision is usually preserved. There are no specific treatment guidelines; management is patient-specific and can include active monitoring, treating infection, immunotherapy, radiotherapy and chemotherapy. Prognosis of lymphoma associated with rheumatic disease is similar to the general population (<5% mortality if detected early).

Evidence is conflicting as to whether anti-TNF agents increase the risk of lymphoma. Determining a causal link has proven difficult, due to the rarity of lymphoma and confounding factors, but mechanisms such as decreased activation of natural killer cells, a key defence against lymphoma, have been proposed.  This contributed to the decision to stop etanercept in our patient.  However, interestingly the lymphoma spontaneously resolved whilst the patient was still taking etanercept, which perhaps argues against the drug being a driving pathogenic factor.

Another consideration in the decision to switch treatment was the potential overlapping benefit of rituximab. Cells of MALT lymphomas express CD20 antigen on their surfaces, and indeed this was observed in our patient’s immunostaining. Such tumours are exquisitely sensitive to rituximab, and consequently it is often used first-line particularly in areas such as the orbit where radiotherapy can be problematic. It was therefore decided that rituximab would be the best treatment to optimise both conditions, despite there being no immediate need for immunological treatment of the lymphoma itself.

**Case report - Key learning points:**

This case highlights the need to be aware of the increased risk of lymphoma in autoimmune rheumatic diseases such as rheumatoid arthritis and Sjogren’s syndrome. The risk is elevated further in patients over 60 and those with chronically active disease. One must be vigilant during clinical assessment as patients often present with localised symptoms only, which could be dismissed as non-significant. In our case, signs and symptoms were limited to the left eye.  Certainly, if any suspicious clinical features are detected, we advise a low threshold for prompt imaging studies followed by biopsy. Most such lymphomas are low-grade with effective treatment options, but do carry a small risk of high-grade transformation. Therefore, early recognition and treatment is vital and can be life-saving.

Use of anti-TNF agents is recommended in patients with risk factors for developing lymphoma, especially given that cumulative inflammatory disease activity itself may represent a greater risk. Nevertheless, a low threshold of suspicion should be maintained. Based on uncertainties in the evidence, current expert consensus is to avoid anti-TNF agents in patients with current malignancy or any prior melanoma, and to exercise caution with a history of malignancy in the last 5 years. The safest course of action after a diagnosis of lymphoma is to stop anti-TNF agents.

We suggest consideration of rituximab as a safe alternative biologic treatment, given its efficacy in both conditions. Here, it was rationalised that using rituximab to treat the patient’s rheumatoid arthritis could have an added protective effect on her risk of lymphoma recurrence. To investigate this theory further, an interesting opportunity for further work would be to observe a cohort of such patients and compare the lymphoma recurrence rates of those who continued anti-TNF treatment against those who switched to rituximab or stopped biologics altogether.

